# DPP3: From biomarker to therapeutic target of cardiovascular diseases

**DOI:** 10.3389/fcvm.2022.974035

**Published:** 2022-10-12

**Authors:** Peng Ye, Wei Duan, Yue-Qi Leng, Yang-Kai Wang, Xing Tan, Wei-Zhong Wang

**Affiliations:** ^1^Department of Marine Biomedicine and Polar Medicine, Naval Medical Center, Naval Medical University (Second Military Medical University), Shanghai, China; ^2^Key Laboratory of Medical Electrophysiology of Ministry of Education, Medical Electrophysiology Key Lab of Sichuan Province, Institute of Cardiovascular Research, Southwest Medical University, Luzhou, China

**Keywords:** dipeptidyl peptidase 3, cardiovascular diseases, biomarker, therapeutic target, renin-angiotensin system

## Abstract

Cardiovascular disease is the leading cause of death globally among non-communicable diseases, which imposes a serious socioeconomic burden on patients and the healthcare system. Therefore, finding new strategies for preventing and treating cardiovascular diseases is of great significance in reducing the number of deaths and disabilities worldwide. Dipeptidyl peptidase 3 (DPP3) is the first zinc-dependent peptidase found among DPPs, mainly distributes within the cytoplasm. With the unique HEXXGH catalytic sequence, it is associated with the degradation of oligopeptides with 4 to 10 amino acids residues. Accumulating evidences have demonstrated that DPP3 plays a significant role in almost all cellular activities and pathophysiological mechanisms. Regarding the role of DPP3 in cardiovascular diseases, it is currently mainly used as a biomarker for poor prognosis in patients with cardiovascular diseases, suggesting that the level of DPP3 concentration in plasma is closely linked to the mortality of diseases such as cardiogenic shock and heart failure. Interestingly, it has been reported recently that DPP3 regulates blood pressure by interacting with the renin-angiotensin system. In addition, DPP3 also participates in the processes of pain signaling, inflammation, and oxidative stress. But the exact mechanism by which DPP3 affects cardiovascular function is not clear. Hence, this review summarizes the recent advances in the structure and catalytic activity of DPP3 and its extensive biological functions, especially its role as a therapeutic target in cardiovascular diseases. It will provide a theoretical basis for exploring the potential value of DPP3 as a therapeutic target for cardiovascular diseases.

## Introduction

Cardiovascular disease is a major cause of disability and premature death worldwide, taking the lives of 17.9 million people in 2019, of which 81% occur in developing countries, and over one-third are premature deaths (1–3). Meanwhile, they impose a tremendous socioeconomic burden on patients and the healthcare system, especially in low-income and middle-income countries (4). Although health consciousness and medical practices have been improved gradually, the treatment and prognosis of cardiovascular diseases remain unsatisfactory (5). Therefore, finding new strategies for preventing and treating cardiovascular disease is of great significance in reducing the number of deaths and disabilities worldwide.

Dipeptidyl peptidases (DPPs) are a class of proteolytic enzyme family involved in nearly all aspects of cellular activities and physiological functions (6). They play a crucial role in a variety of physiological and pathological processes. There are eight distinct subtypes in this family, including DPP1 (7, 8), DPP2 (9, 10), DPP3 (11–13), DPP4 (14–16), DPP6 (17), DPP8 (18, 19), DPP9 (20, 21), and DPP10 (22, 23) (Table 1). DPPs are involved in a variety of physiological processes, including oligopeptide N-terminus processing, bio-active peptide degradation, cell cycle regulation, protein maturation, and viral infection (24–26). Contemporary studies have shown that DPPs inhibitors can be effective in treating several diseases, such as diabetes, tumors, and hematological diseases (27). Among them, DPP4 inhibitors, such as vildagliptin and sitagliptin, can reduce the level of blood glucose by enhancing the effects of the insulin-stimulating peptides glucagon-like peptide-1 (GLP-1) and glucose-dependent insulin-stimulating polypeptide (GIP). Furthermore, this class of drugs is used to treat type 2 diabetes and improve glycemic control (28). Similarly, a non-selective inhibitor of DPP, Val-boroPro (talabostat), can also treat prostate cancer by affecting fibroblast activation protein *via* reducing angiogenesis and inhibiting tumor proliferation and invasion (29). And the activity of DPP2 and the ratio of DPP2/DPP4 in serum may be diagnostic indicators for rheumatoid arthritis, systemic lupus erythematosus, cancer, Parkinson's disease, and other diseases (30, 31).

**Table 1:** DPPs family and functions.

**Name**	**Peptidase activity**	**Physiologic function**	**References**
Dipeptidyl peptidase 1-DPP1	Cysteine hydrolase	Immune responses to bacterial infections and sepsis	(7, 8)
Dipeptidyl peptidase 2-DPP2	Serine peptidase	Oligopeptide hydrolysis	(9, 10)
Dipeptidyl peptidase 3-DPP3	Metal aminopeptidase	Regelation of pain, blood pressure, and oxidative stress	(11–13)
Dipeptidyl peptidase 4-DPP4	Serine peptidase	Glucose homeostasis	(14–16)
Dipeptidyl peptidase 6-DPP6	Serine peptidase	Regulating the expression and activation of potassium ion voltage-gated channel D2 isoforms	(17)
Dipeptidyl peptidase 8-DPP8	Serine peptidase	Immune responses	(18, 19)
Dipeptidyl peptidase 9-DPP9	Serine peptidase	Dipeptide hydrolysis	(20, 21)
Dipeptidyl peptidase 10-DPP10	Serine peptidase	Not clear	(22, 23)

Dipeptidyl peptidase 3 (DPP3), one of the main members of the DPPs family, is highly conserved among animals. Its hydrolysis of 4–10 amino acid residues plays an important part in metabolism (32). The molecular weight of the purified DPP3 homologs is between 69 and 89 kDa (33), with the D. melanogaster DPP3 isoform (82–89 kDa) and cockroach DPP3 isoform (76–80 kDa) also found and verified (34). And DPP3 has multiple isoforms, including the classical DPP3 variant 1 (UniProtKB Q9NY33-1), variant 2 (UniProtKB Q9NY33-2), and variant 4 (UniProtKB Q9NY33-4). Variant 2 has no peptidase activity due to the lack of a catalytic sequence, while variant 4 lacks amino acids 91–120 from variant 1, but still has a catalytic function (35). Since the discovery of DPP3, its role in various physiological and pathological processes has attracted widespread attention from scientists. Studies have found that DPP3 can participate in protein turnover (32), oxidative stress (35, 36), pain (37), ovarian cancer tissue invasiveness (38), colorectal cancer progression (39), the maintenance of bone homeostasis (40) and inflammation (41, 42). Moreover, DPP3 is also closely related to high mortality in patients with sepsis (43, 44), cardiogenic shock (45), and acute kidney injury (46). Especially in cardiovascular diseases, DPP3 is regarded as a marker of more severe disease with higher activity of renin-angiotensin system (RAS) (47). However, there is still a lack of unified understanding of the role of DPP3 in diagnosing and treating cardiovascular diseases. Therefore, this review will summarize the biological characteristics of DPP3 and its research progresses in cardiovascular diseases, which aims to provide a theoretical basis for exploring potential value of DPP3 as a therapeutic target for cardiovascular diseases.

## Biological properties

### Distribution of DPP3

DPP3 is widely distributed among organisms. It was firstly identified in the bovine pituitary in 1967 (48). And this enzyme is the third to be found in the DPPs family, hence naming it DPP3. It can hydrolyze the terminal dipeptidyl amino residue from polypeptides containing at least four residues (49). According to a rat RNA-Seq transcriptomic results across 11 organs and four developmental stages (50, 51), DPP3 can highly expresses in the cardiovascular organs including heart and blood vessels. Moreover, accumulating studies have demonstrated that DPP3 exists in the other cardiovascular related organs, such as the adrenal gland, brain (52), and liver (53). Of course, the expression of DPP3 has also been reported in other organs that are not closely related to cardiovascular function, for instance, red blood cells (54), and cataractous lens (55). In addition, DPP3 has been found to be expressed not only in rats and humans, but also in yeast cells (56) and *Drosophila* (57). In 2000, Abramić et al. (58) found a high similarity and conservation of DPP3 between human erythrocytes and rat liver by mass spectrometry. Similar conclusions have been drawn in other studies (59–61).

DPPs are widely distributed among different tissues and were detected in the blood plasma (62), cerebrospinal fluid (26) and other body fluid (63). However, DPP3 is originally thought to be a cytoplasmic peptidase at the sub-cellular level, because it can be purified from the soluble fraction of most mammalian tissue homogenates (64). After that, DPP3 was extracted from the cell membrane of the Alzheimer's disease mouse model and detected by mass spectrometry, which confirmed that the peptidase is also distributed in mammalian cell membranes (65). Furthermore, DPP3 has also been found in extracellular fluids such as postplacental serum, human seminal, and cerebrospinal fluid, except in cells (66, 67). However, the mechanism of DPP3 targeting cell membranes or secretion into extracellular fluids is unclear. Recently, DPP3 activity was detected in human HK-2 cell culture medium (68), suggesting that intracellular DPP3 may be secreted or released into the extracellular fluids (69). Meanwhile, after cell death mediated by the anti-Fas receptor (CD95) antibody, DPP3 activity was significantly increased in the medium due to disrupting the cytoplasmic membrane (70). As cell death is a significant pathological change in a disease state, intracellular DPP3 can enter the circulation due to massive cell death (71). A recent clinical trial also found a close relationship between progressive cell death and the high level of DPP3 in plasma during the shock of various etiologies (72). It is precisely the wide distribution of DPP3 inside and outside cells that make it participating in various physiological and pathological processes, such as oxidative stress (73–75), RAS over-activation (45, 76), and inflammation (77, 78). Therefore, the above evidence fully indicates that DPP3 is widely distributed in multiple organisms and tissues and may have a broader range of biological functions.

#### Catalytic specificity of DPP3

Although DPP3 is widely distributed, its substrate and catalytic mode have strong specificity (79). DPP3 consists of two lobes separated by a wide clef, one is a α-helix-rich upper lobe, and the other is a lower lobe that mixes α-helices and β sheets (80, 81). Regardless of the fact that enzyme specificity exists between species, additional helical loops of amino acid residues are commonly observed in loops and the surface of both structures (82). In 1999, for the first time (83), it was verified that the DPP3 family (M49 family) has a unique HEXXGH conserved motif, and the catalytic motif (HEXXGH) and the secondary motif (EECRAR/D) are part of the upper lobe, while there are substrate binding sites between the upper and lower lobes. Two histidine residues on this motif contribute to the binding of divalent metal ions (mainly Zn^2+^, partly Mn^2+^, Co^2+^, Ni^2+^, and Cu^2+^). Among them, the Zn^2+^is located at the conserved binding site in the upper part of the two structures. And it is crucial for the catalytic activity of DPP3, where the glutamine acid provides the catalytic base with the histidine residue coordinating Zn^2+^ (84). In addition, DPP3 has a series of conserved arginine called “arginine anchors”, which are located at different positions from the catalytic Zn^2+^ (85). This allows substrates of different lengths to form a salt bridge between their C-terminus and the guanidine group of the positioned arginine anchor, thereby ensuring that the peptide bond cleaved by DPP3 is in the correct position, making DPP3-substrate binding easier (82). DPP3 has a wide range of active sites and flexible conformations, and the binding sites can be adjusted to different lengths and substrate binding, while 4–8 peptides are the most suitable hydrolysis substrates for DPP3. Among many polypeptides, angiotensin II (Ang II), endorphins and enkephalins can be efficiently cleaved by DPP3 (86).

#### DPP3 and enkephalin

Methionine-enkephalin (YGGFM) and leucine-enkephalin (YGGFL) are endogenous opioid neurotransmitters in the brain and spinal cord of many animals, including humans. And G. G. HADDAD and colleagues (87) found enkephalin analogs given intravenously or intra-arterially induce a biphasic response in MAP. While Li et al. (88) demonstrated that increased enkephalin in the rostral ventrolateral medulla after electro acupuncture decreases blood pressure. N terminal of both enkephalin are anchored to DPP3 *via* hydrogen bonding and electrostatic interactions of the tyrosine-318, glutamate-316, and asparagine-394 side chains and cleaved by DPP3 (89). Furthermore, leucine-enkephalin binds to inactive DPP3 isoform, but the difference in the C-terminal residue between the two structures of enkephalin is not significant. According to the results above, DPP3 may play a significant role in the regulation of cardiovascular function through its relationship with enkephalin.

#### DPP3 and endorphin 2

Endorphins are opioid peptides that play an essential role as neurotransmitters or neuromodulators in mammals (90), whose main functions include analgesia and endothelial cell-dependent vasodilation. Endorphins can be divided into two types according to their amino acid composition: Endorphin 1 (YPWF-NH2) and endorphin 2 (YPFF-NH2), both of which have amidated C-terminal (91). Kassab et al. (92) found endorphin is involved in the responses of blood pressure and heart rate to pain in sleep-deprived rats. In the sino-aortic denervated rat, the content of beta-endorphin and leu-enkephalin were decreased in hypothalamus and medulla oblongata (93). As DPP3 has now turned out to be a post-proline peptidase (38), substrates containing proline are more easily cleaved by peptidases. The binding mode of endorphin 2 to DPP3 was demonstrated, which mainly binds and interacts with the conserved residues aspartate-316, asparagine-391, and asparagine-394 of peptidase through the N-terminus with micromolar affinity (94). However, whether DPP3 is involved in the regulation of endorphins on cardiovascular function is still unclear.

#### DPP3 and synthetic morphorphins

Tyrosine (valine-valine-tyrosine-proline-tryptophan), a derivative of rotorphanin (95), has been shown to inhibit the activity of purified DPP3 in the brain of monkey (96). By synthesizing orphanoid pentapeptides containing aliphatic or aromatic amino acids at the N-terminus, such as VVYPW, LVYPW, IVYPW, YVYPW, FVYPW, and WVYPW, it was found that among these pentapeptides, IVYPW is a stronger inhibitor than casomorphin agent, and inhibits the activity of rat DPP3 with nanomolar affinity (42, 97).

#### DPP3 and RAS

Ang II is a potent vasoconstrictor of octapeptides, primarily involved in the humoral regulation of cardiovascular activity. In the treatment of hypertension, angiotensin-converting enzyme (ACE) inhibitors, Ang II type 1 receptor (AT1R) antagonists, and mineralocorticoid receptor antagonists are cornerstones in blocking RAS. Although Ang II is not an opioid peptide, DPP3 can cleave Ang II *in vitro*. Zhang et al. (98) demonstrated that Arg421-Lys423 of DPP3 could form an α-helix with the presence of Ang II. Thus, like other opioid peptides, the binding of Ang II to DPP3 is an endothermic process driven by entropy changes. Ang II forms a cis-peptide in a wheel-like conformation between histidine-6 and proline-7 during hydrolysis (99), meanwhile the binding site of DPP3 can bend to fully accommodate upon binding to Ang II and catalytic substrate. It was found that purified angiotensin-(1–7) [Ang-(1–7)] peptidase and DPP3 exhibited the same Ang-(1–7) hydrolysis profile, and both enzymatic activities were inhibited by the metallopeptidase inhibitor JMV-390 (100). DPP3 can sequentially hydrolyze Ang-(1–7) to Ang-(3–7) and rapidly convert Ang-(3–7) to Ang-(5–7) (101). At the same time, the kinetic analysis showed that the hydrolysis rate of Ang-(3–7) was higher than that of Ang-(1–7), and the Km value of Ang-(3–7) was lower than that of Ang-(1–7). Finally, it was found that chronic treatment of HK-2 cells with 20 nM of JMV-390 decreased intracellular DPP3 activity and increased cellular levels of Ang-(1–7) (69). Therefore, DPP3 can not only cleave Ang II but also participate in the hydrolysis of Ang-(1–7) and Ang-(3–7), thereby affecting the balance between Ang II and Ang-(1–7) in RAS.

### General functions of DPP3

As DPP3 cleaves dipeptides sequentially from the N-terminus of various bioactive peptide substrates, it has a very wide range of biological functions. In 2001, Zhan et al. (32) have found DPP3 participates in the intracellular turnover of proteins. In the same year, DPP3 was found to be able to remove the N-terminal dipeptide from the myotropic neuropeptide proctolin *in vitro* (34). On the basis of DPP3's substrate specificity, recent research indicates that it plays a role in the regulation of blood pressure and pain (102). Since studies identified the DPP3 expression and activity in cells of the innate immune system, such as polymorphonuclear granulocytes and neutrophils, it has been reported that DPP3 involves in regulating the body's immune function (103). What's more, accumulating evidences have demonstrated that the high expression of DPP3 was associated with the pathogenesis of cancers such as multiple myeloma, colorectal cancer, and ER-positive breast cancer (12, 39, 104–106).

## DPP3 and cardiovascular diseases

### DPP3 and hypertension

Current studies have confirmed that abnormally increased Ang II can lead to elevated blood pressure by directly causing vasoconstriction, sympathetic hyperexcitation, and increased aldosterone release (107, 108). Additionally, the rostral ventrolateral medulla and paraventricular nucleus can also produce Ang II that stimulates the Ang II type 1 receptor (AT1R), thereby causing sympathetic excitation to increase blood pressure (109, 110). Not only in the brain, circulatory Ang II can enter the peri-ventricular organs such as subfornical organ and endplate vascular nodes to inhibit the baroreflex activity, resulting in increased vascular tone. And Ang II can also promote a wide range of tissue responses, such as apoptosis, inflammation, and fibrosis through activation of AT1R (111, 112). Therefore, angiotensin-converting enzyme inhibitors and AT1R antagonists, which target Ang II, are currently the first-line treatments for hypertension, especially in patients with hypertension complicated by diabetes or renal function failure (113, 114).

Based on the theory that DPP3, as a highly efficient hydrolase of angiotensin, can participate in the regulation of RAS (115), Xiaoling Pang and colleagues (11) in 2016 found that injecting DPP3 into Ang II-induced hypertensive mice through the tail vein significantly reduced blood pressure. A novel function of DPP3 and its potential therapeutic use in hypertension were revealed for the first time. However, Kumar et al. (86) found that DPP3 knockout mice showed no change in blood pressure using the tail artery cuff method in the same year. Although this study has some limitations due to the use of the tail artery cuff method to measure blood pressure (for example, the effect of stress on blood pressure in mice), the unchanged blood pressure also suggests that there may be other cardiovascular compensatory mechanisms after DPP3 knockout. Angiotensin II, III, and IV can be rapidly removed by exogenous intravenous injection of DPP3, and the dipeptide released by the substrate also has an inhibitory effect on ACE. Therefore, theoretically, DPP3 can lower levels of functional angiotensin, ultimately lowering blood pressure (33, 81). However, further studies are still required to verify whether DPP3 can be used as a biomarker of hypertension or participate in the occurrence and development of hypertension.

### DPP3 and cardiogenic shock

Recent studies have reported that known biomarkers such as brain natriuretic peptide (BNP) (116), N-terminal pro-brain natriuretic peptide (NT-proBNP) (117), growth stimulation expressed gene 2 (ST2) (118), and troponin (119) are not of high value in the prediction of cardiogenic shock, while DPP3 as a recently discovered biomarker is attracting researchers' attention (77, 120). To elucidate the effects of circulating DPP3 on cardiac function and renal hemodynamics, Benjamin Deniau and colleagues (121) measured the circulatory level of DPP3 of 174 patients with acute heart failure and found that a high level of circulatory DPP3 was associated with short-term mortality risk and severe organ dysfunction. Additionally, a rapid decline in the level of DPP3 within 24 h after acute heart failure correlated with a better outcome. In 2020, Dépret et al. (122) also found that the concentration of DPP3 in plasma on admission was closely associated with an increased risk of death and circulatory collapse in severely burned patients. Later, in 2021, Boorsma et al. (47) measured the level of DPP3 in the serum samples of 2,156 patients with acute heart failure using luminescence immunoassay and found that the concentration of DPP3 was increased in patients with worsening heart failure, which may exacerbate acute heart failure. On the contrary, prilizumab, as a specific antibody against DPP3, has a certain potential therapeutic value in patients with acute heart failure. In 2022, Pavo et al. (45) found that the level of circulatory DPP3 was elevated only in patients with advanced heart failure with reduced ejection fraction (HFrEF), which could not only serve as a biomarker of cardiogenic shock, but also help identify end-stage patients with HFrEF. In animal experiments, Deniau et al. (121) also found that intravenous injection of DPP3 in healthy mice can lead to myocardial depression and impaired renal hemodynamics. In contrast, the level of oxidative stress and inflammation was rapidly reduced through injection of procilizumab, an inhibitor of DPP3, which significantly normalized cardiac function and renal hemodynamics in the mouse model of acute heart failure. The above studies show that circulatory DPP3 plays an important role in the diagnosis and staging of patients with cardiogenic shock caused by acute heart failure. However, the mechanism of the elevated level of DPP3 in pathological conditions and whether it is involved in the occurrence and development of cardiogenic shock is still unclear.

### DPP3 and chronic heart failure

Unlike acute cardiogenic shock, the exogenous administration of DPP3 showed a protective effect on the development of myocardial fibrosis and chronic heart failure. Given that excess Ang II can damage organs such as the heart and kidneys, it was found that exogenous intravenous administration of DPP3 for 4 weeks significantly reduced the degree of Ang II-induced cardiac fibrosis and exerted a protective effect (11). At the same time, Komeno et al. (46) also found that the cardiac inflammatory cell infiltration and myocardial fibrosis levels were significantly reduced, and diastolic cardiac dysfunction was also improved after 8 weeks of intravenous recombinant DPP3 treatment in the type 2 diabetes model db/db mice, but it has no significant effects on blood glucose. The above results have shown that DPP3 can prevent the occurrence and development of chronic heart failure by inhibiting myocardial inflammation and fibrosis. Although the specific mechanism of DPP3 protection against chronic heart failure is still unclear, these results suggest that DPP3 may be a potential therapeutic target for cardiovascular diseases.

### Possible mechanisms of DPP3 involved in cardiovascular diseases

#### Imbalance of RAS

RAS is a peptide hormone system composed of various components such as enzymes, inactive peptides, and active peptides, which play an essential role in regulating blood pressure and body fluid homeostasis. Traditionally, angiotensinogen produced in the liver is hydrolyzed by renin from paraglomerular cells to produce angiotensin I (10-peptide), which is then converted by ACE to the biologically active Ang II (8-peptide). Ang II is a highly efficient hydrolysis substrate of DPP3 and is also the principal effector peptide of RAS. It can virtually participate in the functional regulation of most organs, including the heart, kidney, and vascular system, and has crucial pathophysiological significance (123).

Angiotensin-converting enzyme 2 (ACE2) can directly catalyze the hydrolysis of Ang II to generate Ang-(1–7), but Ang-(1–7) has the opposite biological effect to Ang II (124). By binding to Mas receptors, Ang-(1–7) can promote vasodilation, anti-proliferation, and anti-hypertrophy (125). At the same time, Ang-(1–7) can also be cleaved by DPP3 to generate Ang-(3–7). Studies have shown that Ang-(3–7) can promote the release of dopamine and γ-aminobutyric acid in the striatum (126). It plays a vital role in regulating blood pressure in the rostral ventrolateral medulla (127), which suggests that DPP3 may play an important role in treating Parkinson's disease and hypertension, respectively. As research progressed, Blet et al. (43) found that a low level of DPP3 (median 15 ng/mL) was also present in the circulatory system of healthy individuals. And in the pathological process of sepsis-induced multiple organ failure, the higher the circulatory DPP3 on admission is associated with the more prolonged need for supportive therapy such as vasopressors and mechanical ventilation. Meanwhile, DPP3 inhibits the signal transduction of the Ang II-AT1 receptor complex by affecting intracellular G protein-coupled receptor-dependent Ca^2+^ in HEK293T cells (128). This laid the theoretical basis for DPP3 to affect RAS and the hemodynamics and development of cardiovascular diseases.

#### Oxidative stress

Besides the over-activation of RAS, oxidative stress may also be a critical mediator between DPP3 and cardiovascular diseases (129). Increased reactive oxygen species (ROS) can adversely affect cellular molecules, such as DNA, RNA, proteins, lipids, and carbohydrates, leading to cell damage and death (130). In contrast, the cellular antioxidant system [superoxide dismutase (SOD), peroxidase, and antioxidant vitamins] can maintain the balance of these two systems by preventing the accumulation of ROS (131). When there is an imbalance between ROS production and antioxidants, oxidative stress will disrupt redox signaling and further lead to endothelial damage, cardiovascular remodeling, renal dysfunction, sympathetic nervous system excitation, and immune cell activation (132, 133). Under normal physiological conditions, Nuclear factor-erythroid-2-related factor 2 (Nrf2) is a leucine zipper transcription factor located in the cytoplasm that binds to the inhibitor of Nrf2 [INrf2, or Kelch-like ECH-associated protein 1 (KEAP1)], which up-regulates a series of antioxidant enzymes. After oxidative damage or phosphorylation of Nrf2 at the serine 40 site by protein kinase C or phosphatidylinositol 3 kinase, Nrf2 is released from the complex with INrf2 and migrates to the nucleus. Afterward, nuclear Nrf2 up-regulates the expression of antioxidant enzymes by binding to the antioxidant response element (ARE) in its promoter (134). However, it is found that elevated ROS levels can promote DPP3 expression through the transcriptional regulator E26 avian erythroblastosis virus transcription factor-1, and elevated DPP3 can mediate the release and migration of Nrf2 to the nucleus, thereby up-regulating the expression of antioxidant enzymes (131). In 2017, Lu et al. (12) found an interaction between endogenous DPP3 and KEAP1, and hydrogen peroxide can strongly induce the DPP3-KEAP1 interaction. In comparison, DPP3 is required for Nrf2 induction and nuclear accumulation in estrogen receptor-positive MCF7 breast cancer cells (135). In addition, a high level of DPP3 mRNA is associated with an increase in expression of Nrf2 downstream genes and a poor prognosis for estrogen receptor-positive breast cancer. After that, Ren et al. (78) in 2021 also found that DPP3 can protect hippocampus neurons by modulating the neuronal KEAP1/Nrf2 signaling pathway, inhibiting apoptosis, oxidative stress, and inflammation in the pathological state of the cerebral ischemia/reperfusion injury.

#### Inflammation

Inflammation is a protective response of the body to injury or infection, but excessive long-term inflammation can lead to the development of cardiovascular diseases (136, 137). A large amount of evidence shows that long-term abnormal changes in inflammatory cells such as macrophages and immune molecules such as tumor necrosis factor-α (TNF-α) can promote the development of myocardial fibrosis and chronic heart failure. While excessive infiltration of inflammatory cells is associated with acute deterioration of cardiac function (138). In addition, a large number of clinical trials have shown that compared with normotensive patients, the levels of interleukin-6 (IL-6) (103), interleukin-1β (IL-1β) (139), and TNF-α (140, 141) in plasma were higher in hypertensive patients. Some studies have reported that DPP3 also has another function, which is involved in inflammation and immune responses. And DPP3 has been detected in the innate and acquired immune systems, such as granulocytes, monocytes, and lymphocytes (142). Moreover, deletion of DPP3 affects not only the production of proinflammatory cytokines, but also the anti-inflammatory cytokines (82, 143). Up-regulation of proinflammatory cytokines TNF-α, IL-1β, and IL-6 were also observed in DPP3-knockout myeloid cells and macrophages (16). These results suggest that DPP3 may be involved in the initiation and maintenance of immune responses, and it may significantly impact the regulation of immune function.

## Summary

**Figure 1:**
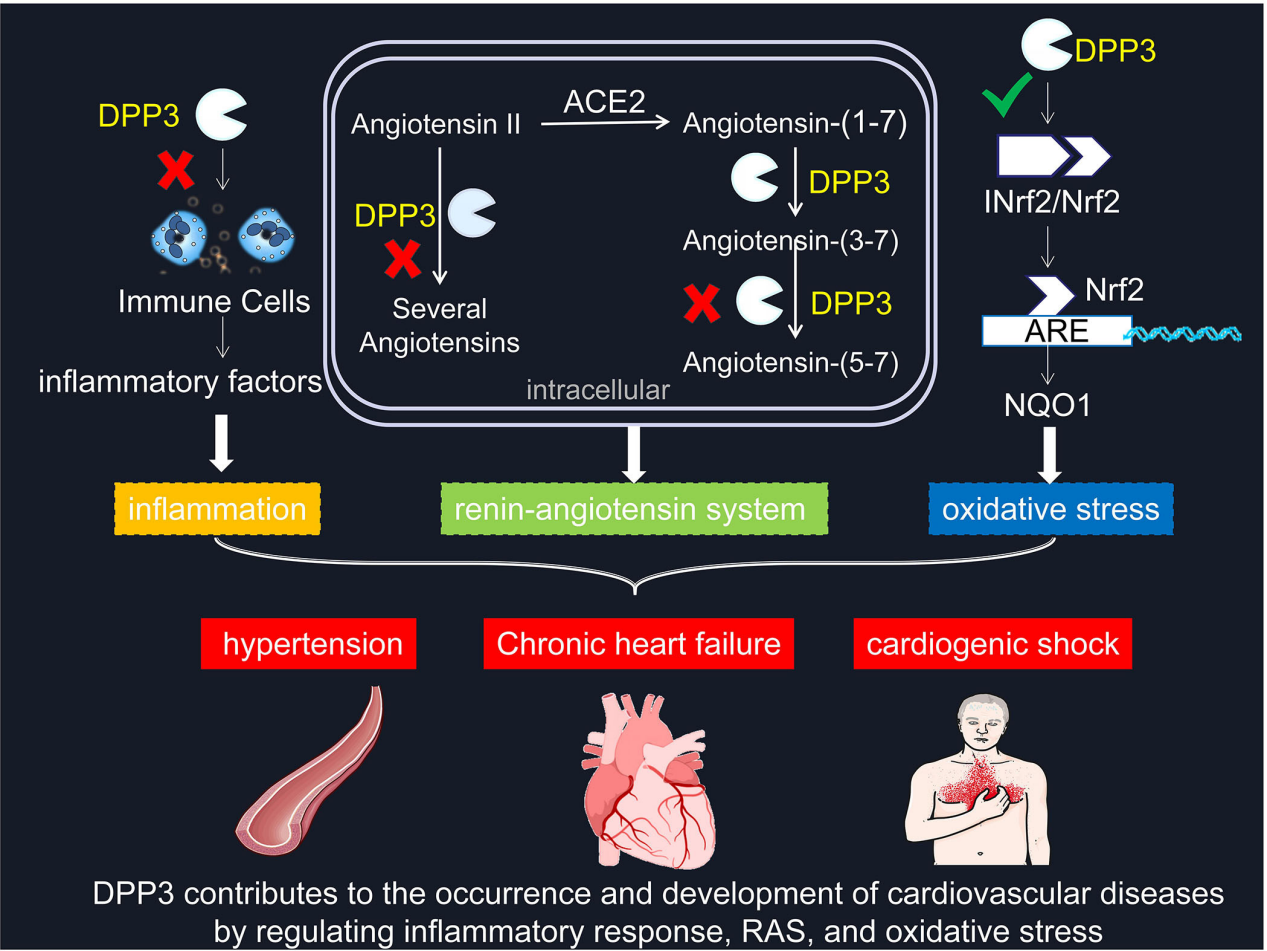
DPP3 contributes to the occurrence and development of cardiovascular diseases by regulating inflammatory response, RAS, and oxidative stress. **×** represents blocking this pathway. **√** means promoting this mechanism. DPP3, Dipeptidyl peptidases 3; RAS, renin-angiotensin system; Nrf2, Nuclear factor-erythroid-2-related factor 2; INrf2, inhibitor of Nrf2; NQO1, quinone oxidoreductase 1.

Globally, cardiovascular diseases are the leading cause of death. More than four-fifths of death in patients with cardiovascular diseases is caused by heart attack or stroke. Hypertension, as an essential risk factor for cardiovascular diseases, substantially increases the risk of cardiovascular and cerebrovascular diseases. Hence, identifying the risk of cardiovascular diseases and ensuring patients receive treatment early are the main strategies to prevent death in patients with cardiovascular diseases. Moreover, the foremost method is the comprehensive use of indicators, including patient history, imaging, and histology. Among them, the diagnostic evaluation of biomarkers in plasma and the targeted administration of cardiovascular drugs are particularly important for accurate diagnosis and prevention of heart attack and stroke. At present, the concentration and activity of DPP3 in circulation can accurately predict the severity of acute cardiogenic shock patients, which undoubtedly provides an important basis for the prevention and diagnosis of cardiovascular diseases. With the in-depth exploration of DPP3, a large amount of evidence shows that it is not only a biomarker of cardiovascular disease, but also participates in the occurrence and development of cardiovascular diseases through pathways such as RAS, oxidative stress, and inflammation. And DPP3 plays an essential role in the pathogenesis of cardiovascular diseases such as hypertension and heart failure (Figure 1), which will contribute to its transformation from biomarker to therapeutic target.

## Perspective

Although the mystery of DPP3 has been gradually unveiled, the results of animal experiments have shown that exogenous intravenous injection of DPP3 inhibitor procilizumab in cardiogenic shock improves cardiac function. In high-risk diseases such as hypertension and diabetes, DPP3 can prevent the occurrence and development of chronic heart failure by inhibiting myocardial inflammation and myocardial fibrosis. The precise mechanism remains elusive for the difference in the role of DPP3 in chronic cardiac failure and cardiogenic shock. At the same time, the level of circulatory DPP3 has been helpful in the diagnosis of diseases such as cardiogenic shock in clinical studies, but whether it can be further used to identify patients sensitive to hemodynamic treatment strategies remains to be explored. Therefore, it is still of great significance to clarify the efficacy of DPP3 as a biomarker and to explore its potential therapeutic value in patients with cardiovascular disease for the diagnosis and prevention of cardiovascular disease.

## Author contributions

PY and WD designed the manuscript. XT and W-ZW edited the manuscript. Y-QL and Y-KW revised the manuscript. All authors contributed to manuscript revision, read, and approved the submitted version.

## Funding

This work was supported by the Natural Science Foundation of Shanghai (No. 22ZR1478400) and the National Natural Science Foundation of China (Nos. 81970354, 81630012, and 81800366).

## Conflict of interest

The authors declare that the research was conducted in the absence of any commercial or financial relationships that could be construed as a potential conflict of interest.

## Publisher's note

All claims expressed in this article are solely those of the authors and do not necessarily represent those of their affiliated organizations, or those of the publisher, the editors and the reviewers. Any product that may be evaluated in this article, or claim that may be made by its manufacturer, is not guaranteed or endorsed by the publisher.
